# Addressing the Peri‐Wound Intact Skin of Hard‐To‐Heal Diabetic Foot Ulcers With Topical Red Deer Conditioned Media (PTT‐6^TM^) Skin Conditioner: A Case Series on Clinical Efficacy

**DOI:** 10.1002/hsr2.70211

**Published:** 2024-11-20

**Authors:** Kimberley Leow, Leon Timothy Charles Alvis, Bradley Edmond Charles Yuen, Leighton Cheng, Shiqi Chiam, Siti Nurfarahdillah Binte Abdul Razak, Gerard Benjamin Evans, Kenneth Koh, Ivor Jiun Lim, Toan Thang Phan, Keng Lin Wong

**Affiliations:** ^1^ Department of Podiatry Sengkang General Hospital Singapore; ^2^ CellResearch Corporation Singapore Singapore; ^3^ Department of Orthopaedic Surgery Sengkang General Hospital Singapore Singapore; ^4^ Department of Orthopaedic Surgery, Yong Loo Lin School of Medicine National University of Singapore Singapore Singapore; ^5^ Musculoskeletal Sciences Academic Clinical Programme Duke‐NUS Medical School Singapore Singapore

**Keywords:** diabetic foot ulcer, mesenchymal stem cells, periwound management, wound healing, wound microenvironment

## Abstract

**Background and Aim:**

The healing of diabetic foot ulcers (DFUs) can be hindered by the susceptibility of the surrounding intact skin to pro‐inflammatory proteases. A conditioned media, known as PTT‐6^TM^, derived from mesenchymal stem cells found in the lining of red deer umbilical cords, has been formulated to protect the intact peri‐wound skin of DFUs. The aim is to evaluate the clinical effectiveness of PTT‐6^TM^ in managing peri‐wound intact skin in hard‐to‐heal DFUs.

**Methods:**

Patients with DFUs that persisted for over 3 months were divided into two subgroups. The active wound group received standard‐of‐care treatment protocol followed by PTT‐6^TM^ application around the peri‐wound area, while the maintenance wound group applied PTT‐6^TM^ media over the healed wound site.

**Results:**

Forty cases were recorded, of which 22 (55%) were included in the active wound group. The majority were male (75%, *n* = 30) and vast majority had cardiovascular risk factors, including diabetes mellitus (100%, *n* = 40), hyperlipidemia (82.5%, *n* = 33), and hypertension (77.5%, *n* = 31). Most patients had forefoot wounds (80%, *n* = 32) on the plantar aspect (82.5%, *n* = 33). The patients in the active wound group had chronic DFUs for a mean of 218 ± 201 days. Of those treated with PTT‐6™ media, 68.4% (*n* = 13) achieved complete wound healing within a mean duration of 69 ± 50 days. Additionally, most patients in the maintenance wound group remained ulcer‐free at 3 months (91.7%, *n* = 11) and 6 months (66.7%, *n* = 6).

**Conclusion:**

The study results suggest that PTT‐6^TM^ media may serve as an additional treatment modality for enhancing the microenvironment at the peri‐wound intact skin site. This could indirectly facilitate wound healing by preserving the integrity of the peri‐wound intact skin.

## 1 Introduction

Diabetic foot ulcers (DFU) are amongst the most severe complications of diabetes mellitus. The lifetime incidence of developing a DFU may be as high as 25% in people with diabetes [1]. Chronic DFUs are associated with an elevated risk of infection and are thus known to be the leading cause of lower limb amputations [2, 3, 4]. As DFU patients spend a considerable amount of time in the hospital and clinic for treatment, the healthcare demands for diabetes have been estimated to cost the Singapore healthcare system SGD 1 billion in 2010 and are expected to increase to SGD 2.5 billion by 2050 [5].

DFUs are difficult to heal and are preceded by major risk factors such as peripheral neuropathy, peripheral arterial disease, foot deformities, plantar pressure overloading, and trauma [6, 7]. A meta‐analysis of DFUs treated in trials with the standard of care revealed a 24% closure rate after 12 weeks [8]. One of the less spoken important aspects of wound healing would be the vulnerable peri‐wound intact skin, that is subjected to physiological impairment and damage, caused by excessive wound exudate, maceration, pressure‐induced callus formation, fragile dry skin, as well as lipid deficiency that lowers skin barrier function which can also compromise wound healing [9]. A negligence of addressing the peri‐wound intact skin in DFU treatment may result in the wound being at risk of further enlargement and infection, which may then delay wound healing.

Studies have reported that hard‐to‐heal DFUs heal poorly due to a prolonged local inflammatory reaction [10, 11, 12]. This is largely due to persistently high levels of pro‐inflammatory cytokines, such as IL‐1B and TNF‐alpha, present in the wound, leading to enhanced protease expression. The most important proteases in disturbed wound healing are matrix‐metalloproteases (MMPs), produced by fibroblasts, keratinocytes, macrophages, and endothelial cells [13]. The MMPs, which are initially beneficial to wound healing to initiate the inflammatory phase, now in excess, cause disruption of the healing process [12, 13]. These proteases, when exposed to the healthy peri‐wound intact skin, may also cause damage to the healthy tissues [10], causing the peri‐wound intact skin to be in a pro‐inflammatory state, disrupting the normal state of physiology, resulting in disturbing healing of these DFUs. Hence, an aspect of peri‐wound intact skin management would be to negate the pro‐inflammatory insults from these proteases.

Current interventions to protect the peri‐wound intact skin include petrolatum, zinc oxide paste or ointments, hydrocolloid dressings, and film‐forming liquid acrylates [11]. These are largely physical barriers that prevent the wound exudates from contacting the peri‐wound intact skin. Hence, it is not surprising that a meta‐analysis conducted by Schuren and colleagues has shown that all the above‐mentioned interventions demonstrated no superiority over each other [11], as none of these current treatments address the potential spill‐over effects of the pro‐inflammatory proteases the wound has on its peri‐wound intact skin.

To address this challenge, a topical skin conditioner derived from red deer umbilical cord lining mesenchymal stem cell (MSC) conditioned media (PTT‐6^TM^) was developed as an adjunctive therapy to protect the integrity of hard‐to‐heal DFU peri‐wound skin [14]. PTT‐6^TM^ is composed of small multicellular segments in specific proportions of cytokines, growth factors, extracellular matrix proteins, amino acids, peptides, and other proteins, which aids in regulating the levels of MMPs [14]. A postulated mechanism would be addressing the underlying pathophysiology of chronic DFUs by promoting fibroblast migration, repressing the proliferation of immune cells in inflammation, and is highly proliferative even under hypoxic conditions [15, 16]. One of the main advantages of using this stem cell skin conditioner is that it may possibly regulate tissue regeneration holistically by optimizing the microenvironment at the peri‐wound intact skin site, thereby promoting wound healing indirectly by ensuring the integrity of the peri‐wound intact skin is not compromised.

In a tertiary hospital care setting, we conducted a series case study to evaluate the clinical effectiveness of a skin conditioner derived from red deer umbilical cord lining MSC conditioned media, PTT‐6™, for the management of peri‐wound intact skin in hard‐to‐heal DFUs. The objectives were to analyze wound healing time and their wound‐free period, in patients with hard‐to‐heal DFUs, after receiving PTT‐6^TM^ for their peri‐wound intact skin as part of their skin care regime.

## 2 Material and Methods

### 2.1 Study Design

This report outlines a series of cases involving patients with hard‐to‐heal DFUs who sought treatment at Sengkang General Hospital, a tertiary healthcare institution in Singapore, between July 2022 and January 2023. Hard‐to‐heal DFUs are defined as patients with a DFU that persisted for more than 3 months despite standard‐of‐care treatment protocols optimized by the Orthopedic Surgery and Podiatry Departments. The standard‐of‐care treatment protocols include local wound care with sharp debridement, dressings promoting a moist wound environment, wound offloading (orthopedic‐grade footwear with custom insoles), and diabetic foot education.

Prior to recruitment, participants were screened for eligibility criteria. To be included in the study, patients had to meet the following criteria: they had either type 1 or type 2 diabetes mellitus, suffered from a hard‐to‐heal DFU, exhibited good vascular status or had peripheral vascular disease and previously undergone vascular intervention to achieve adequate blood flow. Additionally, patients were required to comply with offloading modalities, wound treatment, and clinic appointments. Cases with overt wound infections, ischemia, or noncompliance to offloading and interventions were excluded from the study.

The study population was divided into two subgroups: the active wound group and the maintenance wound group. The active wound group comprised individuals who presented with a hard‐to‐heal DFU, while the maintenance wound group included individuals whose hard‐to‐heal DFU had healed.

The study team monitored and documented relevant baseline patient information, medical history, wound characteristics, and clinical outcomes. All patients provided informed consent for the study, and all patients were part of the hospital's chronic wound registry which received approval from the local institutional review board (reference number: 2020/2854).

### 2.2 Technique Application and Follow‐Up

This study employed the use of PTT‐6^TM^ media, derived from red deer umbilical cord lining MSCs, contained in a 50 mL tube as a skin conditioner. The conditioner was applied to the peri‐wound intact skin surrounding an active wound or over a previously healed wound site. The conditioner was not applied to any open wound.

All patients in the active wound group underwent standard wound care procedures, including wound cleansing and sharps removal of devitalized tissue. Once the wound bed was cleaned, the PTT‐6^TM^ media was applied around the peri‐wound area, precisely 1 cm away from the wound edges, followed by standard‐of‐care dressings to promote moist wound healing. Patients continued with the same offloading modalities as indicated before study enrollment based on their wound characteristics and biomechanics of the foot, which were monitored and optimized accordingly as needed due to wear. The frequency of PTT‐6^TM^ application ranged between 2 and 3 times per week until adequate healing had been achieved. Wound healing was defined as the complete re‐epithelialization of the wound, indicating the restoration of the skin's protective barrier. In case a patient's wound was infected, PTT‐6^TM^ application would be ceased immediately, and the appropriate medical attention would be given, which could include antibiotics prescription, in‐patient admission, and/or surgical intervention, depending on the severity of the infection. These outcomes were duly recorded.

For the maintenance wound group, patients were advised to apply PTT‐6^TM^ media daily, akin to a skin moisturizer, over the healed wound site. They were instructed to apply the media gently, in a circular motion, until it was fully absorbed into the skin. Patients were also encouraged to maintain proper foot hygiene practices and attend follow‐up appointments to monitor their wound‐free period.

### 2.3 Outcome Measures

The active wound group underwent evaluation by assessing the healing of the hard‐to‐heal DFUs, while the maintenance wound group was subjected to continuous monitoring and tracking of their wound‐free period at both 3‐ and 6‐month intervals.

## 3 Results

Baseline demographics, clinical and wound characteristics, and outcomes of both active and maintenance wound patient population were presented in Tables 1 and 2, respectively. In the active wound study cohort, a total of 22 patients were included. A majority were male patients (68.2%, *n* = 15) with a mean age of 57.3 ± 8.3 years old. Cardiovascular risk factors such as suboptimally controlled diabetes mellitus (100%, *n* = 22) with mean HbA1c of 7.7 ± 1.7%, hyperlipidemia (86.4%, *n* = 19) and hypertension (81.8%, *n* = 18) were commonly observed. In terms of wound characteristics, a majority had forefoot wounds (81.8%, *n* = 18) found on the plantar aspect (81.8%, *n* = 18). Prior to the commencement of the study recruitment process, it was observed that the patients had been experiencing chronic DFUs for an average period of 218 ± 201 days. Of the 19 patients with follow‐up data, 68.4% (*n* = 13) achieved wound healing with a mean duration of 69 ± 50 days, while 15.8% (*n* = 3) failed to heal or encountered infection.

**Table 1:** Active wound group.

Variables	*N*	Values
**Baseline demographics**		
Gender, % (*n*)	22	
Male		68.2% (15)
Female		31.8% (7)
Age, mean (SD)		57.3 (8.3)
Race, % (*n*)		
Chinese		40.9% (9)
Malay		27.3% (6)
Indian		22.7% (5)
Others		9.1% (2)
**Comorbidities, % (*n*)**		
DM	22	100.0% (22)
HbA1c, mean (SD)		7.7 (1.7)
HLD		86.4% (19)
HTN		81.8% (18)
CKD		22.7% (5)
PVD		27.3% (6)
IHD		22.7% (5)
Stroke		9.1% (2)
**Wound characteristics**		
Wound location, % (*n*)	22	
Forefoot		81.8% (18)
Midfoot		13.6% (3)
Rearfoot		4.5% (1)
Wound surface, % (*n*)		
Plantar		81.8% (18)
Dorsal		18.2% (4)
Duration of chronic wound (days), mean (SD)		218 (201)
**Outcomes**		
Outcome, % (*n*)	19	
Healed		68.4% (13)
Not healed		15.8% (3)
Infected		15.8% (3)
Duration to healed wound (days), mean (SD)	13	69 (50)
Number of podiatry visits, mean (SD)	19	7.0 (5.6)

**Table 2:** Maintenance wound group.

Variables	*N*	Values
**Baseline demographics**		
Gender, % (*n*)	18	
Male		83.3% (15)
Female		16.7% (3)
Age, mean (SD)		59.1 (9.4)
Race, % (*n*)		
Chinese		50.0% (9)
Malay		33.3% (6)
Indian		16.7% (3)
Others		0.0% (0)
**Comorbidities, % (*n*)**		
DM	18	100.0% (18)
HbA1c, mean (SD)		7.6 (0.9)
HLD		77.8% (14)
HTN		72.2% (13)
CKD		22.2% (4)
PVD		27.8% (5)
IHD		44.4% (8)
Stroke		5.6% (1)
**Wound characteristics, % (*n*)**		
Wound location	18	
Forefoot		77.8% (14)
Midfoot		5.6% (1)
Above ankle		16.7% (3)
Wound surface		
Plantar		83.3% (15)
Dorsal		16.7% (3)
**Outcomes, % (*n*)**		
Outcome at 3 months	12	
Remained healed		91.7% (11)
Recurred		8.3% (1)
Outcome at 6 months	9	
Remained healed		66.7% (6)
Recurred		33.3% (3)

For the maintenance wound study cohort, 18 patients were included with a majority of male patients (83.3%, *n* = 15) and a mean age of 59.1 ± 9.4 years old. Similarly, there was a high proportion of patients with traditional cardiovascular risk factors including diabetes mellitus (100%, *n* = 18), hyperlipidemia (77.8%, *n* = 14), and hypertension (72.2%, *n* = 13). Diabetes mellitus was again suboptimally controlled with a HbA1c of 7.6 ± 0.9%. Notably, a significant proportion of patients also had underlying ischemic heart disease (44.4%, *n* = 8). The wound profile was similar to patients with active wounds, with majority being found on the forefoot (77.8%, *n* = 14) and over the plantar aspect (83.3%, *n* = 15). Outcomes were favorable with a vast majority remaining ulcer‐free at 3 months (91.7%, *n* = 11) and 6 months (66.7%, *n* = 6).

## 4 Discussion

The present study investigates the topical application of PTT‐6^TM^ conditioned media, derived from red deer umbilical cord lining stem cells, as a skin conditioner to enhance the treatment of persistent hard‐to‐heal DFUs in conjunction with standard‐of‐care treatment protocols. Despite the patients having hard‐to‐heal DFUs for an average of 218 ± 201 days, 68.4% of patients in the active wound group went on to achieve wound healing with a mean duration of 69 ± 50 days. Additionally, the majority of patients in the maintenance wound group maintained ulcer‐free status at 3 months (91.7%) and 6 months (66.7%).

These findings highlight the potential of this stem cell skin conditioner in healing impaired wounds complicated by multiple comorbidities. It is hypothesized that the MSC from PTT‐6^TM^ can mitigate the spill‐over effects of pro‐inflammatory proteases from chronic wounds, optimize the microenvironment at the peri‐wound intact skin site, and regulate tissue regeneration [17]. Indirectly, this promotes wound healing by preserving the integrity of the peri‐wound intact skin, which is also pivotal for extending wound‐free periods [18].

The study had documented three patients who experienced DFU infections, but there was no evidence to suggest that the application of PTT‐6^TM^ media was responsible for the infections. The study also found no adverse events associated with the topical application of PTT‐6^TM^ media, suggesting that it is a safe and effective adjunctive treatment for chronic DFUs [19].

The study has several limitations. First, it had a small sample size, thereby reducing the generalizability of our findings. Moreover, the absence of a comparison group makes it challenging to determine the true impact of PTT‐6^TM^ media on regulating peri‐wound impairment and promoting wound healing. To determine the statistical and clinical significance of PTT‐6^TM^ media in healing chronic DFUs, a more extensive study with a randomized controlled trial or a comparison group would be necessary, along with mechanism studies to prove the efficacy of PTT‐6^TM^ media in hard‐to‐heal DFU peri‐wound intact skin. Long‐term studies and observations will also be necessary to investigate the long‐term effects of PTT‐6^TM^ media.

## 5 Conclusion

The present findings suggest that the utilization of PTT‐6^TM^ could potentially serve as a valuable adjunct to the standard‐of‐care management of hard‐to‐heal DFUs, leading to potentially improved clinical outcomes and decreased healthcare expenses in the long run. However, it remains imperative to conduct further rigorous investigation and validation of this therapy through randomized controlled trials.

## Author Contributions

**Kimberley Leow:** data curation, formal analysis, investigation, methodology, project administration, resources, software, supervision, validation, writing–original draft, writing–review and editing. **Leon Timothy Charles Alvis:** project administration, resources, supervision, writing–review and editing. **Bradley Edmond Charles Yuen:** investigation, methodology. **Leighton Cheng:** investigation, methodology. **Shiqi Chiam:** investigation, methodology. **Siti Nurfarahdillah Binte Abdul Razak:** investigation, methodology. **Gerard Benjamin Evans:** investigation, methodology. **Kenneth Koh:** investigation, methodology. **Ivor Jiun Lim:** funding acquisition, resources, writing–review and editing. **Toan Thang Phan:** funding acquisition, resources, writing–review and editing. **Keng Lin Wong:** conceptualization, funding acquisition, investigation, methodology, project administration, resources, supervision, visualization, writing–review and editing. All authors have read and approved the final version of the manuscript.

## Conflicts of Interest

Keng Lin Wong receives honorarium as a speaker for CellResearch Corporation, 3M‐KCI Medical Asia Pte. Ltd., Urgo Medical APAC, and Molnlycke Health Care Asia‐Pacific Pte. Ltd. Ivor Jiun Lim and Toan Thang Phan are associated with CellResearch Corporation, which is known to own intellectual properties related to Cord Lining Stem Cells technology and is a provider of PTT‐6^TM^ for the study. The two authors state that the manuscript was written without any undue influence from them and without any attempt to inject opinions that will benefit the technology. The other authors declare no conflicts of interest.

### Transparency Statement

The lead author Kimberley Leow affirms that this manuscript is an honest, accurate, and transparent account of the study being reported; that no important aspects of the study have been omitted; and that any discrepancies from the study as planned (and, if relevant, registered) have been explained.

## Data Availability

The data that support the findings of this study are available from the corresponding author, Kimberley Leow, upon reasonable request. Kimberley Leow, the corresponding author, had full access to all of the data in this study and takes complete responsibility for the integrity of the data and the accuracy of the data analysis.

## References

[1] K. L. Andrews , M. T. Houdek , and L. J. Kiemele , “Wound Management of Chronic Diabetic Foot Ulcers: From the Basics to Regenerative Medicine,” Prosthetics & Orthotics International 39, no. 1 (February 2015): 29–39. 25614499 10.1177/0309364614534296

[2] C. M. Capobianco and J. J. Stapleton , “Diabetic Foot Infections: A Team‐Oriented Review of Medical and Surgical Management,” Diabetic Foot & Ankle 1, no. 1 (January 2010): 5438. 10.3402/dfa.v1i0.5438 PMC3284273 22396806

[3] V. Nube , T. Bolton , E. Chua , and D. Yue , “Osteomyelitis in the Diabetic Foot: What Lies Beneath,” Primary Intention: The Australian Journal of Wound Management 15, no. 2 (2007): 49.

[4] A. Denjalić , H. Beculić , A. Jusic , and L. Bečulić , “Evaluation of the Surgical Treatment of Diabetic Foot,” Medicinski Glasnik 11 (August 2014): 307–312. 25082245

[5] M. E. Png , J. Yoong , T. P. Phan , and H. L. Wee , “Current and Future Economic Burden of Diabetes Among Working‐Age Adults in Asia: Conservative Estimates for Singapore From 2010–2050,” BMC Public Health 16, no. 1 (February 2016): 153. 26880337 10.1186/s12889-016-2827-1 PMC4754926

[6] B. J. Earl , P. A. Lazzarini , E. M. Kinnear , and P. L. Cornwell , “Prevalence of Active Foot Disease and Foot Disease Risk Factors in a Subacute Inpatient Rehabilitation Facility: A Cross‐Sectional Prevalence Study,” Journal of Foot and Ankle Research 7, no. 1 (December 2014): 41. 25328541 10.1186/s13047-014-0041-x PMC4200129

[7] I. Dietrich , G. A. Braga , F. G. de Melo , and A. C. C. da Costa Silva Silva , “The Diabetic Foot as a Proxy for Cardiovascular Events and Mortality Review,” Current Atherosclerosis Reports 19, no. 11 (November 2017): 44. 28971322 10.1007/s11883-017-0680-z

[8] D. J. Margolis , J. Kantor , and J. A. Berlin , “Healing of Diabetic Neuropathic Foot Ulcers Receiving Standard Treatment. A Meta‐Analysis,” Diabetes Care 22, no. 5 (May 1999): 692–695. 10332667 10.2337/diacare.22.5.692

[9] A. Rowledge , N. Frescos , C. Miller , E. Perry , and W. McGuiness , “The Diabetic Foot Ulcer Periwound: A Comparison of Visual Assessment and a Skin Diagnostic Device,” Wound Practice & Research: Journal of the Australian Wound Management Association 24, no. 3(2016): 160–168.

[10] K. F. Cutting and R. J. White , “Maceration of the Skin and Wound Bed. 1: Its Nature and Causes,” Journal of Wound Care 11, no. 7 (July 2002): 275–278, 10.12968/jowc.2002.11.7.26414. 12192848

[11] J. Schuren , A. Becker , and R. Gary Sibbald , “A Liquid Film‐Forming Acrylate for Peri‐Wound Protection: A Systematic Review and Meta‐Analysis (3M Cavilon No‐Sting Barrier Film),” International Wound Journal 2, no. 3 (September 2005): 230–238, 10.1111/j.1742-4801.2005.00131.x. 16618326 PMC7951666

[12] R. Lobmann , G. Schultz , and H. Lehnert , “Proteases and the Diabetic Foot Syndrome: Mechanisms and Therapeutic Implications,” Diabetes Care 28, no. 2 (February 2005): 461–471, 10.2337/diacare.28.2.461. 15677818

[13] M. Motzkau , J. Tautenhahn , H. Lehnert , and R. Lobmann , “Expression of Matrix‐Metalloproteases in the Fluid of Chronic Diabetic Foot Wounds Treated With a Protease Absorbent Dressing,” Experimental and Clinical Endocrinology & Diabetes 119, no. 5 (May 2011): 286–290, 10.1055/s-0030-1267235. 21031342

[14] C. Ong , I. Lim , M. Goldman , N. Hartman , J. Masilamani , and T. Phan , “Improvement in Skin Elasticity Using Red Deer Umbilical Cord Lining Mesenchymal Stem Cell Conditioned Media,” Journal of Drugs in Dermatology 22, no. 1 (January 2023): 82–89, 10.36849/JDD.6906. 36607757

[15] S. N. Blumberg , A. Berger , L. Hwang , I. Pastar , S. M. Warren , and W. Chen , “The Role of Stem Cells in the Treatment of Diabetic Foot Ulcers,” Diabetes Research and Clinical Practice 96, no. 1 (April 2012): 1–9. 22142631 10.1016/j.diabres.2011.10.032

[16] S. Hendrawan , Y. Kusnadi , C. A. Lagonda , et al., “Wound Healing Potential of Human Umbilical Cord Mesenchymal Stem Cell Conditioned Medium: An in Vitro and in Vivo Study in Diabetes‐Induced Rats,” Veterinary World 14, no. 8 (August 2021): 2109–2117. 34566328 10.14202/vetworld.2021.2109-2117 PMC8448625

[17] P. D. Huynh , Q. X. Tran , S. T. Nguyen , V. Q. Nguyen , and N. B. Vu , “Mesenchymal Stem Cell Therapy for Wound Healing: An Update to 2022,” Biomedical Research and Therapy 9, no. 12 (2022): 5437–5449, 10.15419/bmrat.v9i12.782.

[18] S. Kanji and H. Das , “Advances of Stem Cell Therapeutics in Cutaneous Wound Healing and Regeneration,” Mediators of Inflammation 2017 (2017): 1–14, 10.1155/2017/5217967. PMC5682068 29213192

[19] A. Musiał‐Wysocka , M. Kot , and M. Majka , “The Pros and Cons of Mesenchymal Stem Cell‐Based Therapies,” Cell Transplantation 28, no. 7 (2019): 801–812, 10.1177/0963689719837897. 31018669 PMC6719501

